# Antibody Responses to Crude Gametocyte Extract Predict *Plasmodium falciparum* Gametocyte Carriage in Kenya

**DOI:** 10.3389/fimmu.2020.609474

**Published:** 2021-02-03

**Authors:** Brian R. Omondi, Michelle K. Muthui, William I. Muasya, Benedict Orindi, Ramadhan S. Mwakubambanya, Teun Bousema, Chris Drakeley, Kevin Marsh, Philip Bejon, Melissa C. Kapulu

**Affiliations:** ^1^Department of Biosciences, KEMRI-Wellcome Trust Research Programme, Kilifi, Kenya; ^2^Department of Biochemistry and Molecular Biology, Egerton University, Nakuru, Kenya; ^3^Department of Medical Microbiology, Radboud University Medical Centre, Nijmegen, Netherlands; ^4^Department of Infection Biology, London School of Hygiene & Tropical Medicine, London, United Kingdom; ^5^Centre for Tropical Medicine and Global Health, Nuffield Department of Clinical Medicine, University of Oxford, Oxford, United Kingdom

**Keywords:** *Plasmodium falciparum*, gametocytemia, gametocyte extract, antibody response, malaria transmission

## Abstract

**Background:**

Malaria caused by *Plasmodium falciparum* remains a serious global public health challenge especially in Africa. Interventions that aim to reduce malaria transmission by targeting the gametocyte reservoir are key to malaria elimination and/or eradication. However, factors that are associated with gametocyte carriage have not been fully explored. Consequently, identifying predictors of the infectious reservoir is fundamental in the elimination campaign.

**Methods:**

We cultured *P. falciparum* NF54 gametocytes (to stage V) and prepared crude gametocyte extract. Samples from a total of 687 participants (aged 6 months to 67 years) representing two cross-sectional study cohorts in Kilifi, Kenya were used to assess IgG antibody responses by ELISA. We also analyzed IgG antibody responses to the blood-stage antigen AMA1 as a marker of asexual parasite exposure. Gametocytemia and asexual parasitemia data quantified by microscopy and molecular detection (QT-NASBA) were used to determine the relationship with antibody responses, season, age, and transmission setting. Multivariable logistic regression models were used to study the association between antibody responses and gametocyte carriage. The predictive power of the models was tested using the receiver operating characteristic (ROC) curve.

**Results:**

Multivariable logistic regression analysis showed that IgG antibody response to crude gametocyte extract predicted both microscopic (OR=1.81 95% CI: 1.06–3.07, *p*=0.028) and molecular (OR=1.91, 95% CI: 1.11–3.29, *p*=0.019) *P. falciparum* gametocyte carriage. Antibody responses to AMA1 were also associated with both microscopic (OR=1.61 95% CI: 1.08–2.42, *p*=0.020) and molecular (OR=3.73 95% CI: 2.03–6.74, *p*<0.001) gametocytemia. ROC analysis showed that molecular (AUC=0.897, 95% CI: 0.868–0.926) and microscopic (AUC=0.812, 95% CI: 0.758–0.865) multivariable models adjusted for gametocyte extract showed very high predictive power. Molecular (AUC=0.917, 95% CI: 0.891–0.943) and microscopic (AUC=0.806, 95% CI: 0.755–0.858) multivariable models adjusted for AMA1 were equally highly predictive.

**Conclusion:**

In our study, it appears that IgG responses to crude gametocyte extract are not an independent predictor of gametocyte carriage after adjusting for AMA1 responses but may predict gametocyte carriage as a proxy marker of exposure to parasites. Serological responses to AMA1 or to gametocyte extract may facilitate identification of individuals within populations who contribute to malaria transmission and support implementation of transmission-blocking interventions.

## Introduction

Malaria remains a serious public health challenge globally, especially within the tropics. The disease results in over 200 million clinical cases and over 400,000 deaths each year with infants and pregnant mothers in Africa bearing the greatest burden (1). Key challenges that have hampered the elimination of this parasitic infection have been lack of an effective vaccine and the emergence of drug-resistant parasites (2). Interventions that lower transmission by reducing the gametocyte reservoir within the human host or inhibiting parasite development within the vector are considered key to malaria elimination (3).

Gametocytes are the sexual, non-pathogenic, and non-replicative form of *P. falciparum* parasites responsible for malaria transmission (4). They are produced when a fraction, usually less than one-tenth, of asexually replicating parasites commit to sexual development (5). Within the human host, gametocytes develop over five distinct morphological stages (I–V), a process that takes between 9 and 12 days (6). Immature gametocytes (stage I–IV) are found sequestered away in the bone marrow and spleen with only the mature stage V being present in peripheral circulation (7–9). Previous studies have demonstrated that mature stage V gametocytes, which are taken up by the mosquito vector during a blood meal, comprise less than 5% of the total parasite biomass (5, 10, 11).

Both symptomatic and asymptomatic infections have been associated with gametocyte carriage (12). Notably, a huge proportion of gametocytes are sub-microscopic, with molecular methods being an order of magnitude more sensitive than microscopy (13–15). This has important implications for malaria elimination efforts as malaria transmission has been observed even at sub-microscopic gametocytemia (16, 17). Identifying individuals with sub-microscopic carriage is fundamental in defining the infectious reservoir. As molecular parasite detection methods may not always be available in the field, other prognostic indicators are required. In addition, gametocyte densities tend to fluctuate over time thus lower densities at a given time-point may be misleading if higher densities occurred earlier. Previous studies have identified anemia (4), high asexual parasitemia (4, 18, 19), and young host age (19, 20) as key factors associated with gametocyte carriage. Serological markers have also been associated with gametocytemia (21). However, factors that are associated with gametocytemia have not been fully explored (22). Identification of these factors is important as this can aid the identification of groups that significantly contribute to transmission thus facilitating the implementation of malaria transmission-blocking interventions (18, 23–25). Primaquine administration is recommended to reduce malaria transmission in low transmission areas (26) as a strategy for the reduction of malaria transmission. Therefore, predictors of gametocyte carriage can help identify where to focus such mass drug administration program (MDA) in the fight against the disease.

In this study, we sought to develop an ELISA-based assay as a tool to identify gametocyte carriers. We utilized gametocyte cultured parasites to prepare the antigen to detect IgG antibody responses that would serve as a marker for exposure. Antibody response to blood-stage antigen apical membrane antigen 1 (AMA1) was also explored. We examined the association between these antibody responses to both molecular and microscopic gametocyte carriage in a cohort of naturally exposed individuals living in an endemic area in Kilifi, Kenya.

## Methods

### Study Design and Data Collection

Data and samples from participants recruited and consented to participate in two cross-sectional cohorts: assessment of the infectious reservoir of malaria (AFIRM) (11) and Kilifi malaria longitudinal cohort (KMLC) (18, 27, 28) were included in this study (**Table 1**). These participants were recruited from the Kenyan Coast, Kilifi County, in the sub-locations of Junju and Ngerenya. Malaria transmission in the study area is low to moderate, with transmission intensity going up in the rainy season (May–December) relative to the dry months (January–April) (29).

**Table 1:** Distribution of study participants by demographic characteristics.

Study cohort	AFIRM			KMLC		
**Study location**	Junju			Ngerenya		Junju
**Sub-group**	NA			Early	Late	NA
**Duration of study**	May–Dec 2014	Jan–Apr 2014		1998–2001	2002–2016*	2007–2016
**Season**	Wet	Dry		NA	NA	NA
**Number of Participants**	274	139†		50	127	97
**Age (in years) %, (n/N)**						
**<5**	24.1% (66/274)	11.5% (16/139)		48.0% (24/50)	42.5% (54/127)	41.2% (40/97)
**5–9**	13.5% (37/274)	10.8% (15/139)		42.0% (21/50)	42.5% (54/127)	39.2% (38/97)
**10–15**	14.2% (39/274)	12.9% (18/139)		10.0% (5/50)	15.0% (19/127)	19.6% (19/97)
**>15**	48.2% 132/274	64.8% (90/139)		NA	NA	NA
**Gender, % Female (n/N)**	60.5% (166/274)	31.0% (96/139)		50.0% (25/50)	31.5% (40/127)	67.0% (65/97)
**Asexual parasite positive % (n/N)**						
** Microscopy**	17.2% (47/274)	6.5% (9/139)		78.0% (39/50)	18.7% (24/127)	59.8% (58/97)
**18s QT** **NASBA**	50.4% (138/274)	35.3% (49/139)		NA	NA	NA
**Gametocyte positive % (n/N)**						
** Microscopy**	2.2% (6/274)	1.4% (2/139)		50.0% (25/50)	6.3% (8/127)	35.1% (34/97)
**Pfs25 QT NASBA**	29.6% (81/274)	17.9% (25/139)		NA	NA	NA

^†^35 participants recruited in January–February 2015; *no cross-sectional survey conducted in 2006; NA refers to not applicable.

In the AFIRM cohort, children (<5 years), school-age children (5–15 years), and adults (>15 years) were recruited from Junju regardless of parasite status. The study excluded those who had serious clinical conditions requiring immediate medical attention. A total of 413 participants were recruited over a period of 14 months (between January 2014 and February 2015) and sampling was done both during the dry and wet seasons (**Table 1**) (11).

For the KMLC cohort, a subset of study participants comprising children (<5 years) and school-age children (5–15 years) recruited between 1998 and 2016 from Junju and Ngerenya were included in this study (18). Participants from Ngerenya were initially recruited at random from the community whereas the Junju cohort was selected from children who had initially taken part in a malaria vaccine trial (28). The subset included in this study was identified by first selecting all gametocyte-positive participants during the study period (1998–2016). Thereafter we selected two sets of control groups. The first control group consisted of asexual parasite positive but gametocyte negative participants while the second group had both asexual negative and gametocyte negative participants as determined by microscopy. This gave a total of 1,092 possible samples for analysis out of which only 274 samples had corresponding sera samples available for analysis. Thus, we included a total of 274 participants from the KMLC cohort. In our analysis, we divided the Ngerenya cohort into Ngerenya early (1998–2001) and Ngerenya late (2002–2016) to account for the decline in malaria transmission during the stated period (**Table 1**) (18).

### Ethics Statement

The parent studies were approved by the Kenya Medical Research Institute Ethics Review Committee (reference numbers KEMRI/SERU/SSC2574—AFIRM cohort; and KEMRI/SERU/CGMRC//3149 and KEMRI/SERU/SSC1131—KMLC cohort). All the participants, their parents and/or guardians (in the case of those below 18 years) provided written informed consent and Principles of the Helsinki Declaration were observed during the conduct of the studies.

### Parasite Detection

For microscopic parasite detection, data were available and analyzed for both the KMLC and AFIRM cohorts. In the KMLC cohort, microscopy was the only method used to detect malaria parasites as previously described (18). Briefly, blood films were fixed with methanol (100%) and afterward stained for 45 min with Giemsa (3%). For thick films, asexual parasite count was based on the number of parasites per 200 white blood cells (WBCs) whereas the count was determined per 500 red blood cells for thin films (18). In the AFIRM cohort, parasite density was determined by assuming 8000 WBCs per microliter of blood (11). Gametocytes were observed alongside the asexual parasites and two independent microscopists determined the readings with a third microscopist ascertaining discordant results.

Molecular assay data were available for the AFIRM cohort and methods used previously described (11). Briefly, blood samples were collected by venipuncture and subsequently an automated QIAextraction (Qiagen, UK) was used to extract DNA from 100µl of whole blood samples for qPCR asexual parasite detection. For assays based on RNA, 100µl of whole blood was stored in appropriate volume of TRIzol (Invitrogen, UK) and extraction was done as previously described (15). Estimation of asexual parasite burden was undertaken using either 18S rRNA quantitative nucleic acid sequence-based amplification (QT-NASBA) or qPCR (18s qPCR) for *P. falciparum* asexual parasite detection. For quantification of *P. falciparum* gametocytes, Pfs25 mRNA QT-NASBA was used as previously described (15).

### Parasite Culture and Production of Gametocyte Extract

*Plasmodium falciparum* NF54 gametocytes were cultured as previously described (30) with some modifications. Incomplete culture media was prepared by adding 5.96g/l HEPES (Gibco, UK), 1.96g/l glucose (Sigma, UK), 200mM L-glutamine (Invitrogen, UK), 50mg/l hypoxanthine (Sigma, UK), and 40mg/l gentamicin (Invitrogen, UK) to 10.4g RPMI 1640 (Gibco, UK). Albumax II (Gibco, UK) was added to a final concentration of 10% to produce complete culture media. Asexual parasites were cultured in O^+^ red blood cells at conditions of 92% N_2_, 3% O_2_, 5% CO_2_, and 37°C. The parasites were maintained at 5% hematocrit and 6% parasitemia. Synchronization by repeated sorbitol treatment was performed (0–6 and 18–24 h post-invasion) using 5% D-sorbitol (Sigma, UK) on the cycle preceding gametocyte induction when the parasites were 5% rings (day-1). This was to ensure that the majority of the asexual parasites committed to sexual development at a relatively similar time and thus we could have a large proportion of mature gametocytes at harvest. On the day of induction (day 0), the parasitemia was at 10% rings, this was subsequently diluted to 1%. Afterwards, washed blood cells were added to the culture to attain 5% hematocrit at 30ml culture volume. This was followed by changing the media from day one onward by replacing ¾ of spent media with warm fresh media. From day 3 onwards, the temperature was maintained at 37°C during media change to prevent premature activation as the gametocytes matured. Gametocyte development was monitored by making 10% Giemsa smears and harvesting was done on day 13 post-induction when the parasites were between stages IV and V.

At harvest, the culture was at 3% gametocytemia (**Supplementary Figure 1**). This was centrifuged at 1,800rpm for 5 min to remove culture media. The pellet was subsequently diluted in a ratio of 1:5 in carbonate bicarbonate buffer and sonicated for 30 min. This was followed by three cycles of freeze-thawing (exposure to −80°C for 10 min followed by rapid thawing at room temperature (RT) for 10 min). The extract was then stored at −80°C until use.

### Enzyme-Linked Immunosorbent Assay

We measured antibody responses to crude gametocyte extract and the blood-stage antigen AMA1. As gametocytes are a progeny of asexual parasites (5), exposure to asexual parasites would likely be an indicator of gametocytemia (4, 18, 19). Recombinant AMA1 has been previously used as a marker of asexual parasite exposure (31). We used AMA1 as a known and validated marker of asexual parasite exposure to characterize whether antibody responses to crude gametocyte extract would be a marker for gametocyte carriage.

ELISA was performed as described elsewhere (20) with some modifications in the measurement of human IgG responses to crude gametocyte extract and AMA1. Briefly, 100µl of gametocyte extract (diluted 1:250 in carbonate bicarbonate buffer) and AMA1 (used at a concentration of 0.5μg/ml) were individually coated onto MaxiSorp ELISA plates (Thermo Fisher, UK) and incubated at 4°C overnight. The plates were then washed four times in 1x PBS (Oxoid, UK) containing 0.05% Tween 20 detergent (VWR, UK). This was followed by blocking using 200µl blocking buffer (5% skimmed milk diluted in 1x PBS-0.05% Tween 20) for 2 h at RT. Washing was done as described above. 100µl of sera samples (diluted 1:500 in blocking buffer) was added to the wells followed by incubation for 2 h at RT (gametocyte extract) and overnight at 4°C (AMA1). Eight malaria-naive serum samples and a pool of serum from gametocyte-positive individuals were included as negative and positive controls, respectively. The gametocyte-positive serum control was pooled using equal amounts of serum (100µl per participant) from 80 participants in the AFIRM cohort who had the highest detectable gametocytemia by molecular testing (Pfs25 QT NASBA). Known concentrations of malaria immunoglobulin (MIG) were titrated on each plate to generate a standard curve for extrapolation of antibody units. One hundred microliters of polyclonal rabbit IgG/HRP (Dako, UK) diluted 1:5,000 in blocking buffer was added to the wells followed by incubation for 1 h at RT and washed as earlier described. Subsequently, 100µl of O-phenylenediamine dihydrochloride (OPD) substrate (Sigma, UK) was added and incubation was done for 15 min in the dark. The reaction was then stopped by adding 25 µl of 2M H_2_SO_4_ and absorbance read at 492nM. Sera samples were analyzed in duplicate.

### Statistical Analysis

All analyses were performed using R Version 3.6.1 (32). We summarized the prevalence of asexual parasitemia and gametocytemia by season, transmission setting (location), and age (aggregated into distinct age groups). Wilcoxon test was used to compare age-specific crude gametocyte extract antibody response, AMA1 antibody response, gametocytemia, and asexual parasitemia. This test was also used to compare anti-gametocyte extract IgG antibody response and gametocytemia across season and location of varying transmission intensity. We also estimated the Spearman correlation between crude gametocyte extract response and asexual parasitemia, gametocytemia, and anti-AMA1 antibody response. Correlation was also performed between gametocytemia and asexual parasitemia. Univariable and multivariable logistic regression models were used to study the association between gametocyte carriage and the covariates asexual parasitemia, IgG antibody response to gametocyte extract, IgG antibody response to AMA1, season, and age. Asexual parasitemia was included as a binary variable. The strength of the observed associations was then quantified using odds ratios (OR). Predictive ability of the final model was evaluated using the area under the receiver operating characteristic (ROC) curve (denoted AUC) (33). The AUC ranges from 0.5 (i.e., the null value) to 1. The higher the value above 0.5, the better the predictive power of the model. All tests were performed at 5% significance level where *p < 0.05, **p < 0.01, ***p <0.001, and ****p < 0.0001 were considered significant.

## Results

### Parasitology

Of the total number of study participants under consideration, 25.8% (177/687) were positive for asexual parasites by microscopy while 10.9% (75/687) were positive for gametocytes (**Table 1**). The majority of participants from the KMLC cohort had microscopically detectable asexual parasites, 44.2% (121/274) while 24.2% (67/274) had microscopic gametocytemia. On the other hand, 45.3% (187/413) of participants in the AFIRM cohort had asexual parasitemia by molecular detection (18s QT NASBA) while 13.56% (56/413) had microscopic asexual parasites. Moreover, 25.7% (106/413) of the AFIRM cohort were gametocyte-positive by molecular testing (Pfs25 QT NASBA) while 1.9% (8/413) had microscopically detectable gametocytes.

**Figure 1:**
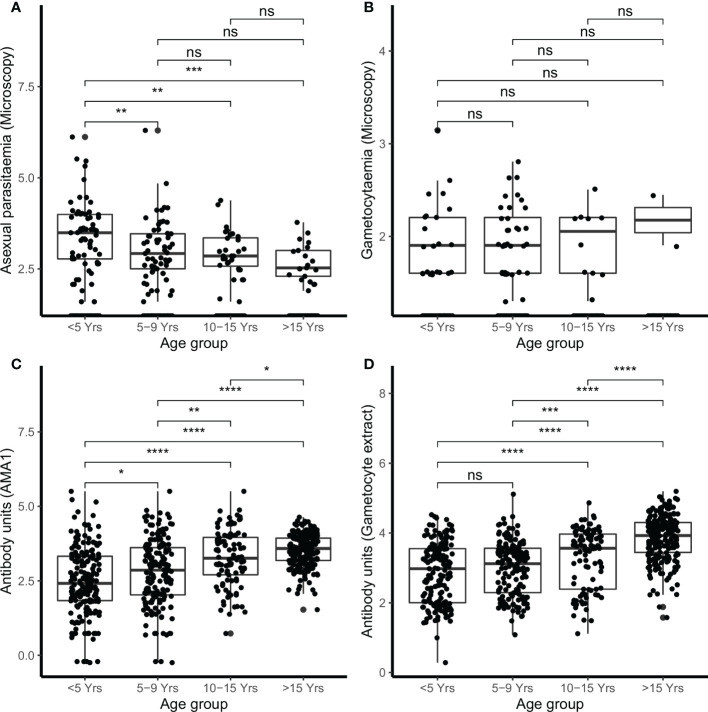
Parasitemia and IgG antibody responses in the combined cohorts by age. Parasites were detected by microscopy **(A)** asexual parasites and **(B)** gametocytes. IgG antibody responses were measured by ELISA **(C)** AMA1 **(D)** gametocyte extract. Box-whisker plots indicate median, minimum and maximum with individual measurements represented by a dot. Shown are combined data from both the Kilifi malaria longitudinal cohort (KMLC) and assessment of the infectious reservoir of malaria (AFIRM) cohorts. Wilcoxon test was used to test significance which is shown and was considered at *p*=0.05 where *p < 0.05, **p < 0.01, ***p < 0.001, ****p < 0.0001, and ns is not significant.

In the combined cohort analysis, younger children (<5 years) had higher densities of microscopic asexual parasitemia compared to children either 5–9 years of age (*p*<0.010), children 10–15 years (*p*<0.010), and adults (>15 years) (*p*<0.001) (**Figure 1A**). However, there were no significant differences in microscopic gametocytemia (**Figure 1B**).

In the AFIRM cohort, children under the age of 5 years had higher microscopic asexual parasite densities (median=3981.07, IQR=6.31) compared to both children aged 5–15 years (median=630.96, IQR=7.94) (*p*<0.010) and adults (>15 years) (median=316.23, IQR=5.01) (*p*<0.001) (**Supplementary Figure 2A**). However, microscopic gametocyte density was not significantly different in the AFIRM cohort (**Supplementary Figure 2B**). School-going children (5–15 years) had higher densities of asexual parasitemia by molecular testing (18s QT NASBA) (median=15.85, IQR=79.43) compared to adults (>15 years) (median=3.98, IQR=63.09) (*p*<0.050) (**Supplementary Figure 2C**), however, there was no significant difference in the densities of molecular detectable gametocytemia (Pfs25 QT NASBA (**Supplementary Figure 2D**).

Of the participants recruited over the dry and wet seasons, 6.5% (9/139) and 17.2% (47/274) respectively in the AFIRM cohort were positive for asexual parasites by microscopy (**Table 1**). Nonetheless, there was no difference between the two seasons regarding microscopic asexual parasitemia (*p*=0.170) (**Supplementary Figure 3A**). In contrast, using a more sensitive assay for detection (18s QT NASBA), there were more participants who were asexual parasite positive in the wet season (50.5%, 138/274) compared to the dry season (35.3%, 49/139) despite there being no observed difference in the density of asexual parasitemia between the two seasons (*p*=0.220) (**Supplementary Figure 3B**). Of the AFIRM cohort participants recruited during the dry season, 17.9% (25/139) had gametocytemia (Pfs25 QT NASBA) by molecular detection while 1.4% (2/139) had microscopically detectable gametocytes. On the other hand, 29.6% (81/274) and 2.2% (6/139) were positive for sub-microscopic (Pfs25 QT NASBA) and microscopic gametocytemia respectively during the rainy season. There was no significant difference in both microscopic (*p*=0.490) (**Supplementary Figure 3C**) and molecular (Pfs25 QT NASBA) (*p*=0.064) (**Supplementary Figure 3D**) gametocytemia densities.

In the KMLC cohort, children under the age of 5 years had higher microscopic asexual parasite densities (median=2511.89, IQR=19.95) compared to children aged 5–9 years (median=1,000.00, IQR=10.00) (*p*<0.050) (**Supplementary Figure 4A**) but there was no observed difference in microscopic gametocyte density (**Supplementary Figure 4B**). Moreover, we observed lower density of asexual parasites among participants recruited from a low transmission setting (Ngerenya Late) (mean=3.16, SD=15.85) compared to a moderate transmission setting, Junju (mean=79.43, SD=50.12) (*p*<0.001) and Ngerenya Early (mean=501.19, SD=39.81) (*p*<0.001) (**Supplementary Figure 4C**). This trend was also true for gametocytemia where Ngerenya Late participants (mean=1.26, SD=3.16) had lower densities than Junju (mean=5.07, SD=10.00) (*p*<0.001) and Ngerenya Early (mean =10.00, SD=10.00) (*p*<0.001) (**Supplementary Figure 4D**).

### IgG Antibody Responses to Gametocyte Extract

To determine sera reactivity against the crude gametocyte extract generated, we measured sero-reactivity by ELISA. The gametocyte-positive serum control (pooled from 80 participants who had the highest molecular detectable gametocytemia in the AFIRM cohort) showed high seropositivity to the gametocyte extract (12 replicates, mean=35,504.00, SD=6,607.00 antibody units).

**Figure 2:**
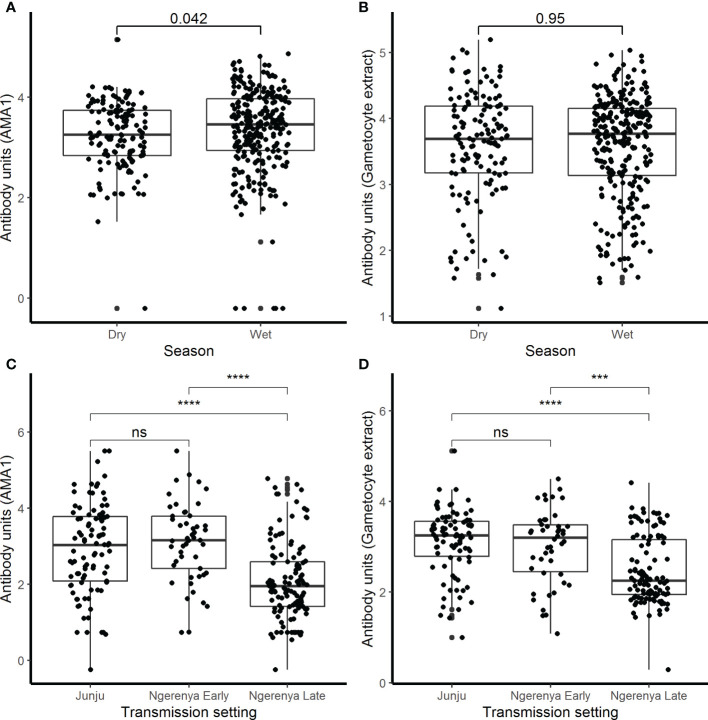
IgG antibody responses by season and transmission setting. IgG antibody responses were measured by ELISA against **(A)** AMA1 and **(B)** crude gametocyte extract shown by season in the assessment of the infectious reservoir of malaria (AFIRM) cohort while against **(C)** AMA1 and **(D)** crude gametocyte extract by transmission setting in the KMLC cohort. Box-whisker plots indicate median, minimum, and maximum with individual measurements represented by each dot. Wilcoxon test was used to test significance which is shown and was considered at p=0.05 where ***p <0.001, ****p < 0.0001, and ns is not significant.

There were 78.2% (537/687) participants who were IgG-positive to the crude gametocyte extract and this included 91.1% (376/413) of participants in the AFIRM cohort and 65.0% (161/244) in the KMLC cohort. In the combined cohort analysis (AFIRM and KMLC), we observed an age-dependent increase in IgG antibody responses to crude gametocyte extract, which was also observed for antibody responses to AMA1 (**Figures 1C, D**). Adults and school-aged children had higher anti-gametocyte extract responses compared to children under the age of 5 years in both cohorts. We also observed significantly higher AMA1 antibody responses during the rainy season (**Figure 2A**) in the AFIRM cohort but only slightly higher responses against the crude gametocyte extract (*p*=0.950) (**Figure 2B**). Interestingly, IgG antibody responses to both the crude gametocyte extract and AMA1 were lower in participants in the KMLC cohort sampled in a low transmission setting (Ngerenya Late) *versus* those from moderate transmission settings; Ngerenya Early (*p*<0.001) and Junju (*p*<0.001) (**Figures 2C, D**).

### Parameters Associated with Crude Gametocyte Antibody Responses

**Figure 3:**
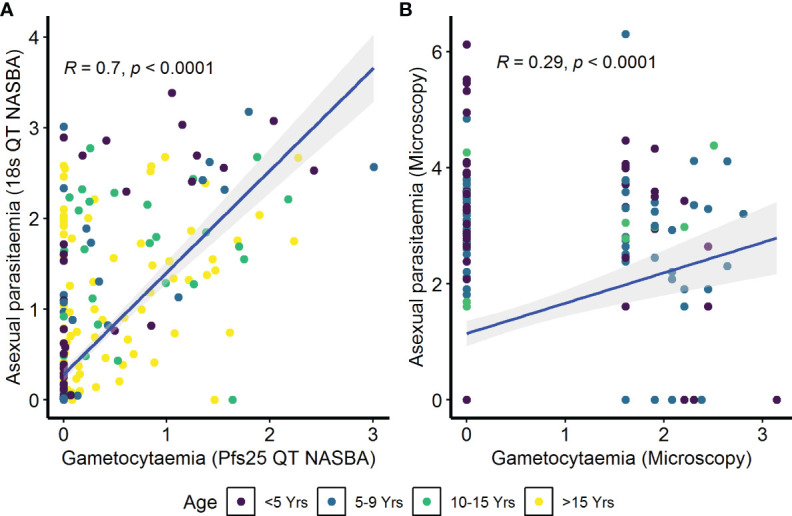
Correlation between asexual parasitemia and gametocytemia. Parasites were detected by **(A)** molecular methods (asexual parasitemia—18s QT NASBA, gametocytes—Pfs25 QT NASBA) in the assessment of the infectious reservoir of malaria (AFIRM) cohort or **(B)** microscopy in the Kilifi malaria longitudinal cohort (KMLC) cohort. Individual dots in the scatter plots represent corresponding measurements from the same participant while regression lines (blue) shows line of best fit and confidence interval are shown in gray.

To determine whether antibody responses against gametocyte extract were associated with the parameters previously identified to be important for carriage, we performed a series of analyses. Initially, we sought to determine the relationship between asexual parasitemia and gametocytemia. In the AFIRM cohort, we observed a positive correlation between gametocytaemia (Pfs25 QT NASBA) and asexual parasitaemia (18s QT NASBA) (ρ=0.70, *p*<0.001) (**Figure 3A**). The same relationship was observed between microscopic gametocytemia and asexual parasitemia in the KMLC cohort (ρ=0.29, *p*<0.001) (**Figure 3B**).

**Figure 4:**
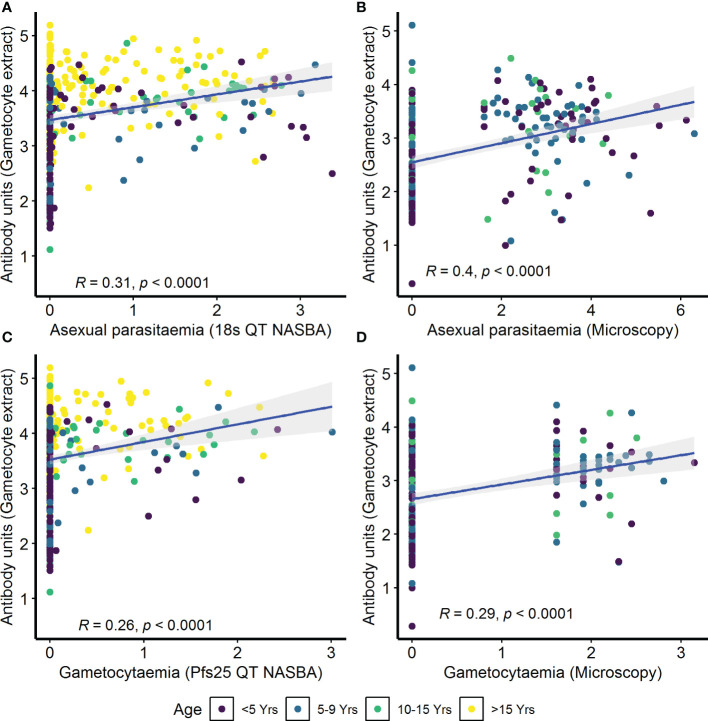
Correlation between parasitemia and crude gametocyte IgG antibody responses. Asexual parasitemia was measured either by molecular detection (18s QT NASBA) in the **(A)** assessment of the infectious reservoir of malaria (AFIRM) cohort or **(B)** microscopy in the Kilifi malaria longitudinal cohort (KMLC) cohort. Gametocytes were detected by either **(C)** molecular methods (Pfs25 QT NASBA) in AFIRM cohort or **(D)** microscopy in KMLC. IgG antibody responses to crude gametocyte extract were measured by ELISA. Individual dots in the scatter plot indicate corresponding measurements from the same study participant and regression lines (blue) show line of best fit and confidence interval is shown in gray.

IgG antibody responses to crude gametocyte extract were positively correlated with both asexual parasitemia as measured by 18s QT NASBA in the AFIRM cohort (ρ=0.31, *p*<0.001) (**Figure 4A**) and asexual parasitemia measured by microscopy in the KMLC cohort (ρ=0.40, *p*<0.001) (**Figure 4B**). Similarly, gametocytemia measured by Pfs25 QT NASBA (ρ=0.26, *p*<0.001) (**Figure 4C**) in the AFIRM cohort and gametocytaemia by microscopy in the KMLC cohort (ρ=0.29, *p*<0.001) (**Figure 4D**) were both positively correlated with IgG antibody responses to crude gametocyte extract. Thus, participants who had high-density asexual parasitemia and gametocytemia also had a high concentration of antibodies to crude gametocyte extract.

**Figure 5:**
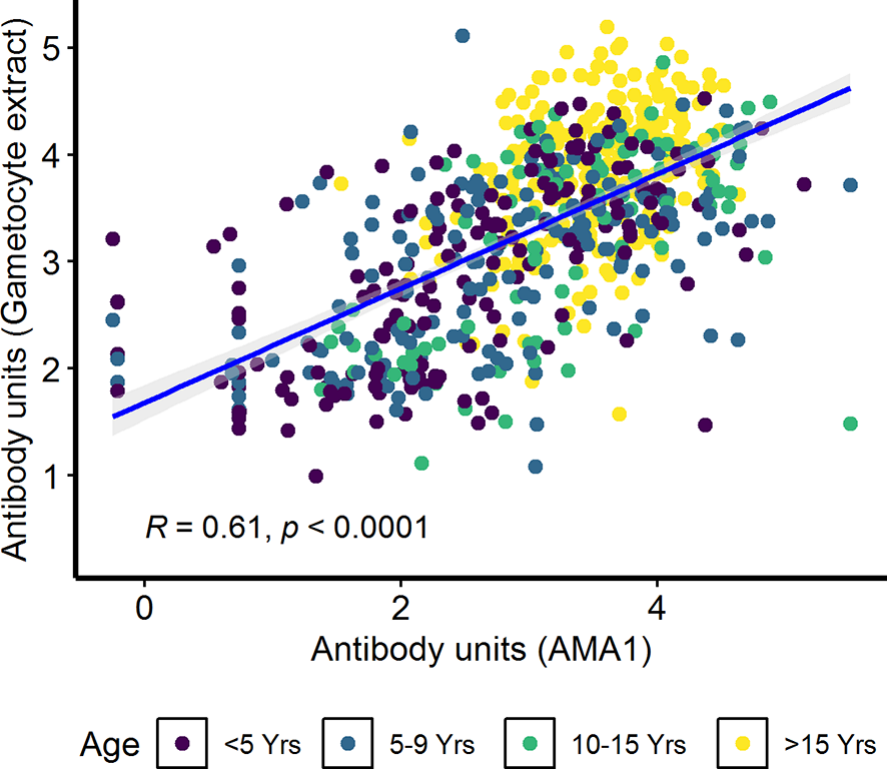
Correlation between antibody responses to AMA1 and crude gametocyte extract. IgG antibody responses to crude gametocyte extract and AMA1 were measured by ELISA. Individual dots in the scatter plot indicate corresponding measurements from the same study participant and regression line (blue) shows line of best fit and confidence intervals are shown in grey. Shown are combined data from both the Kilifi malaria longitudinal cohort (KMLC) and assessment of the infectious reservoir of malaria (AFIRM) cohorts.

In the combined cohort analysis (AFIRM and KMLC), gametocytemia, as measured by microscopy, was positively correlated with asexual parasitemia by microscopy (ρ=0.37, *p*<0.001) (**Supplementary Figure 5A**). On the contrary, there appeared to be no relationship between IgG antibody response to crude gametocyte extract with both asexual parasitemia (ρ=0.04, *p*=0.260) (**Supplementary Figure 5B**) and gametocytemia (ρ=−0.04, *p*=0.340) (**Supplementary Figure 5C**) measured by microscopy was not significant. On the other hand, antibody responses to crude gametocyte extract were highly correlated to the antibody response to AMA1 (ρ=0.61, *p*<0.001) (**Figure 5**) among all the participants from both the KMLC and AFIRM cohorts.

### Predicting Gametocyte Carriage

We tested whether the covariates asexual parasitemia, IgG response to crude gametocyte extract, season, IgG response to AMA1, transmission intensity, and age were predictive of gametocyte carriage. We developed three gametocytemia prediction models; AFIRM molecular model (**Table 2**); KMLC microscopic model (**Table 3**); and combined (AFIRM and KMLC) microscopic model (**Supplementary Table 1**). In the univariable analysis, we observed a significant association between gametocyte carriage and asexual parasitemia, IgG antibody to gametocyte extract IgG, IgG antibody to AMA1, season, age, and transmission intensity.

**Table 2:** Assessment of the infectious reservoir of malaria (AFIRM) cohort molecular model—predictors of molecular gametocytemia (adjusted for gametocyte extract).

Covariate	Univariable analysis			Multivariable analysis		
** **	Odds ratio	95% CI	*p value*	Odds ratio	95% CI	*p value*
** Age group**						
**<5 years**	1.00	–	–	1.00	–	–
**5–15 years**	2.72	1.36–5.44	**0.005**	3.26	1.32–8.05	**0.010**
**>15 years**	1.48	0.77–2.87	0.240	1.29	0.55–3.02	0.560
**Asexual parasitemia**						
**Negative**	1.00	_	**_**	1.00	_	**_**
**Positive**	91.12	27.97–297.04	**<0.001**	75.64	23.10–248.78	**<0.001**
**Gametocyte extract response**	2.52	1.79–3.55	**<0.001**	1.91	1.11–3.29	**0.019**
**Season**						
**Dry**	1.00	–	**-**	1.00	–	–
**Wet**	1.91	1.15–3.17	**0.012**	1.42	0.71–2.87	0.321

*The p-values in bold are statistically significant at level p < 0.05.

**Table 3:** Kilifi malaria longitudinal cohort (KMLC) cohort microscopic model—predictors of microscopic gametocytemia (adjusted for gametocyte extract).

Covariate	Univariable analysis			Multivariable analysis		
** **	Odds ratio	95% CI	*p value*	Odds ratio	95% CI	*p value*
**Age group**						
**<5 years**	1.00	–	–	1.00	–	–
**5–9 years**	1.25	0.66–2.37	0.490	1.18	0.55–2.52	0.670
**10–15 years**	0.95	0.39–2.32	0.910	0.71	0.24–2.13	0.540
**Asexual parasitemia**						
**Negative**	1.00	–	–	1.00	–	–
**Positive**	5.80	2.98–11.28	**<0.001**	2.24	1.02–4.92	**0.044**
**Gametocyte extract response**	2.69	1.78–4.09	**<0.001**	1.81	1.06–3.07	**0.028**
**Cohort**						
**Junju**	1.00	–	–	1.00	–	–
**Ngerenya Early**	2.03	0.97–4.28	**0.061**	2.04	0.86–4.82	0.102
**Ngerenya Late**	0.13	0.05–0.32	**<0.001**	0.23	0.09–0.60	**0.003**

*The p-values in bold are statistically significant at level p < 0.05.

From the multivariable analysis, asexual parasitemia was a key predictor of gametocyte carriage irrespective of the model tested: AFIRM molecular model (OR=75.64, 95% CI: 23.10–248.78, *p*<0.001) (**Table 2**); KLMC microscopic model (OR=2.24, 95% CI: 1.02–4.92, *p*=0.044) (**Table 3**); and combined microscopic (AFIRM and KMLC) model (OR=8.17, 95% CI: 4.25–15.69, *p*<0.001) (**Supplementary Table 1**). IgG antibody responses to crude gametocyte extract were equally observed to be predictive of gametocyte carriage with the odds of being gametocyte-positive increasing approximately two-fold with every 10-fold increase in the antibody concentration in both AFIRM molecular (OR=1.91, 95% CI: 1.11–3.29, *p*=0.019) and KMLC microscopic (OR=1.81, 95% CI: 1.06–3.07, *p*=0.028) models. On the other hand, the anti-crude gametocyte extract responses were not predictive of gametocyte positivity in the combined cohort (AFIRM and KMLC) microscopic model (OR=1.25, 95% CI: 0.87–1.80, *p*=0.230) (**Supplementary Table 1**). This was probably as a result of the low microscopic gametocyte prevalence observed in the AFIRM cohort (1.94%, 8/413) (**Table 1**).

In multivariable models adjusted for AMA1 (without crude gametocyte extract), IgG response to AMA1 was associated with both microscopic (OR=1.61 95% CI: 1.08–2.42, *p*=0.020) (**Supplementary Table 2**) and molecular (OR=3.73 95% CI: 2.03–6.74, *p*<0.001) (**Supplementary Table 3**) gametocytemia. This association was likely due to the strong correlation between asexual parasitemia and gametocytemia (**Figure 3**). When we adjusted for both IgG antibody response to AMA1 and crude gametocyte extract, IgG antibody response to crude gametocyte extract was not predictive of gametocytemia in both the AFIRM molecular model (OR=1.38, 95% CI: 0.77–2.45, *p*=0.278) (**Table 4**) and KMLC microscopic model (OR=1.91, 95% CI: 1.41–2.41, *p*=0.209) (**Table 5**). On the other hand, AMA1 response was predictive of gametocyte carriage when we adjusted for both AMA1 and gametocyte extract in the AFIRM molecular model (OR=3.38, 95% CI: 1.87–6.13, *p*<0.001) (**Table 4**) but not in the KMLC microscopic model (OR=1.53, 95% CI: 0.98–2.42, *p*=0.062) (**Table 5**).

**Table 4:** Assessment of the infectious reservoir of malaria (AFIRM) cohort molecular model—predictors of molecular gametocytemia (adjusted for both AMA1 and gametocyte extract).

Covariate	Univariable Analysis			Multivariable Analysis		
** **	Odds ratio	95% CI	*p value*	Odds ratio	95% CI	*p value*
** Age group**						
**<5 years**	1.00	–	–	1.00	–	–
**5–15 years**	2.72	1.36–5.44	**0.005**	2.36	0.97–5.75	**0.057**
**>15 years**	1.48	0.77–2.87	0.240	0.92	0.39–2.21	0.859
**Asexual parasitemia**						
**Negative**	1.00	_	**_**	1.00	–	**-**
**Positive**	91.12	27.97–297.04	**<0.001**	68.54	20.66–227.36	**<0.001**
**AMA1 response**	5.18	3.24–8.27	**<0.001**	3.38	1.87–6.13	**<0.001**
**Gametocyte extract response**	2.52	1.79–3.55	**<0.001**	1.38	0.77–2.45	0.278
**Season**						
**Dry**	1.00	–	**-**	1.00	–	–
**Wet**	1.91	1.15–3.17	**0.012**	1.10	0.52–2.36	0.796

*The p-values in bold are statistically significant at level p < 0.05.

**Table 5:** Kilifi malaria longitudinal cohort (KMLC) cohort microscopic model—predictors of microscopic gametocytemia (adjusted for both AMA1 and gametocyte extract).

Covariate	Univariable analysis			Multivariable analysis		
** **	Odds ratio	95% CI	*p value*	Odds ratio	95% CI	*p value*
**Age group**						
**<5 years**	1.00	–	–	1.00	–	–
**5–9 years**	1.25	0.66–2.37	0.490	0.61	0.18–2.06	0.427
**10–15 years**	0.95	0.39–2.32	0.910	1.12	0.50–2.52	0.777
**Asexual parasitemia**						
**Negative**	1.00	–	–	1.00	–	–
**Positive**	5.80	2.98–11.28	**<0.001**	1.26	0.51–3.10	0.608
**AMA1 response**	2.06	1.56–2.73	**<0.001**	1.53	0.98–2.42	0.062
**Gametocyte extract response**	2.69	1.78–4.09	**<0.001**	1.41	0.82–2.41	0.209
**Cohort**						
**Junju**	1.00	–	–	1.00	–	–
**Ngerenya Early**	2.03	0.97–4.28	**0.061**	0.99	0.84–4.69	0.116
**Ngerenya Late**	0.13	0.05–0.32	**<0.001**	0.18	0.06–0.51	**0.001**

*The p-values in bold are statistically significant at level p < 0.05.

Young age was predictive of gametocyte carriage in the AFIRM molecular model, with the odds of being gametocyte-positive being higher among children aged 5–15 years (OR=3.26, 95% CI: 1.32–8.05, *p*=0.01) compared to adults (>15 years) (OR=1.29, 95% CI: 0.55–3.02, *p*=0.560) (**Table 2**). We also observed a strong association between gametocyte carriage and transmission intensity. Among the participants from a low transmission setting, Ngerenya Late, an individual was less likely to be gametocyte-positive (OR=0.23, 95% CI: 0.09–0.60, *p*=0.003) compared to participants from a moderate transmission setting, Ngerenya Early (OR=2.04, 95% CI: 0.86–4.82, *p*=0.102) (**Table 3**). Individuals sampled in the wet season were more likely to be gametocyte-positive in the univariable analysis, however, this trend was lost in the multivariable analysis (OR=1.42, 95% CI: 0.71–2.87, *p*=0.321).

**Figure 6:**
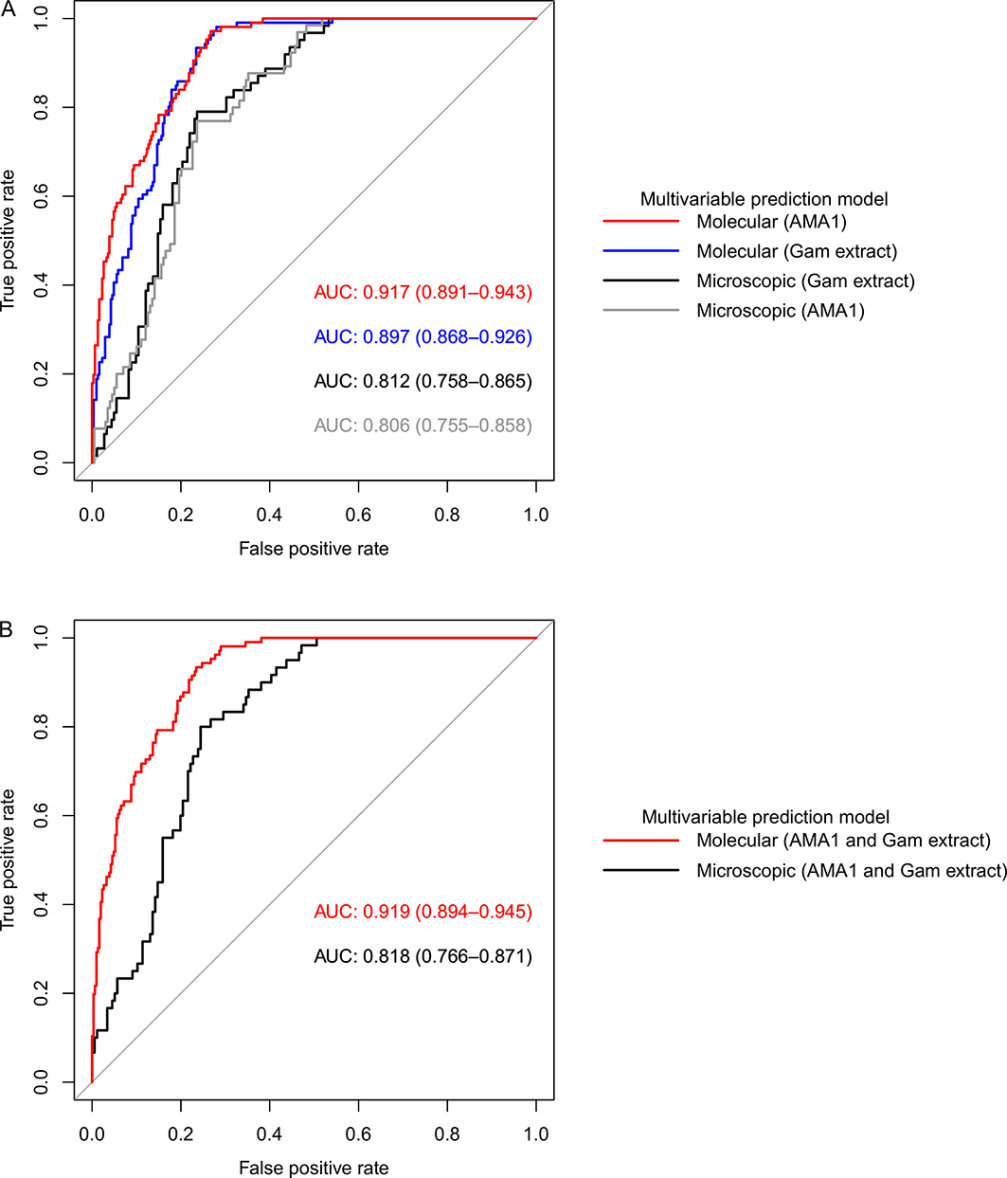
Receiver operating characteristic (ROC) curves showing the predictive power of molecular and microscopic gametocyte carriage prediction models. **(A)** ROC curves for multivariable models adjusted for AMA1 and gametocyte extract independent of each other and **(B)** multivariable models adjusted for both AMA1 and gametocyte extract. The predictive power was measured by area under curve (AUC).

Overall, our models adjusted for gametocyte extract showed very high predictive power for both AFIRM molecular (AUC=0.897, 95% CI: 0.868–0.926) and KMLC microscopic (AUC=0.812, 95% CI: 0.758–0.865) models. Multivariable models adjusted for AMA1 were equally highly predictive in the AFIRM molecular (AUC=0.917, 95% CI: 0.891-0.943) and KMLC microscopic (AUC=0.806, 95% CI: 0.755-0.858) models. In addition, prediction models adjusted for both AMA1 and gametocyte extract were also highly predictive both in AFIRM molecular (AUC=0.919, 95% CI: 0.895-0.945) and KMLC microscopic (AUC=0.818, 95% CI: 0.766–0.871) models (**Figure 6**).

## Discussion

Gametocyte carriage, both microscopic and sub-microscopic, is an important determinant of malaria transmission (18, 19, 22, 25, 34). As in previous studies (13–15), our analysis indicates higher gametocyte prevalence by molecular detection compared to microscopy. This is an important aspect since it has been shown that malaria transmission does occur even at submicroscopic gametocytemia (16, 17). In our findings, age did not appear to impact the density of gametocytes in both cohorts. Previous studies have reported higher densities of gametocytes in children who also tend to have higher asexual parasite densities (22, 34, 35). Nevertheless, the relatively similar densities of gametocyte carriage in our study setting may suggest that older populations also contribute to the gametocyte reservoir. The contribution of all age groups to the infectious reservoir would therefore require that interventions to interrupt malaria transmission such as transmission-blocking vaccines or drugs, be administered to all age groups in an area of active transmission. Where this may prove challenging financially or logistically, targeted application of the transmission-blocking intervention at identified hotspots could be carried out (11, 18).

High prevalence of seropositivity to crude gametocyte extract was observed across all age groups in both cohorts confirming the immunogenicity of the extract. Crude schizont extract has been previously used to screen for malaria exposure for asexual stages (31, 36) thus providing a rationale for measurement of gametocyte carriage using crude gametocyte extract. Interestingly, we noted an age-dependent increase in IgG antibody response to crude gametocyte extract, perhaps suggesting cumulative acquisition of naturally acquired immunity to gametocyte-specific antigens as has been shown for blood-stage antibody responses (37–39). An important finding in this study is that the antibody responses to crude gametocyte extract were associated with gametocyte carriage. However, anti-AMA1 antibodies were either a stronger independent predictor of gametocytemia or equally predictive as anti-gametocyte extract antibodies. The strong association between anti-AMA1 responses and gametocyte carriage is likely because of shared antigens between asexual and sexual stages (21, 40) as gametocytes develop from a subset of asexual parasites (5). On the other hand, the inability of anti-gametocyte extract antibodies in the combined cohort analysis to detect gametocytes could be due to the fact that microscopy does not adequately detect gametocyte carriage especially in asymptomatic individuals (11, 24).

In both the molecular and microscopic gametocyte prediction models, the odds of being gametocyte-positive increased approximately two-fold with every 10-fold increase in IgG antibody concentration to crude gametocyte extract. This could probably explain why we observed lower anti-gametocyte extract responses in a low transmission setting (Ngerenya Late). This preliminary evidence supports the use of crude gametocyte extract ELISA to predict gametocyte exposure. Moreover, since the application of serological biomarkers prepared from recombinant protein, such as recombinant AMA1 used in screening for asexual exposure is relatively more expensive (31) owing to the production of recombinant proteins, the development of crude extracts such as gametocyte lysate, are a viable alternative. There is necessity for point of need (PON) gametocyte diagnostic tests (41) and we propose that ELISA-based crude gametocyte extract assay could potentially serve as an immunoepidemiology test which can be easily adapted and implemented in a number of laboratories. This could identify individuals who require interventions such as drug administration and/or vector control measures to limit transmission.

Interestingly, a previous study exploring gametocyte surface antigen (GSA) responses in a Ghanaian cohort found no association with gametocyte carriage (42). However, this study focused on the surface antigens of immature gametocyte infected red blood cell and not the mature stage V extract which may explain this discrepancy. Moreover, a recent study found an association between microscopic gametocyte carriage and five gametocyte specific antigens (21). Antibody responses to stage V gametocyte infected erythrocyte surface antigens have been previously reported (43), however, this was not observed in a recent study investigating a Malawian cohort (40). Our study focused on the stage V gametocyte extract and not stage V GSAs which may partly explain the observed differential findings. The lysis of stage V gametocyte infected red blood cells to produce the extract potentially releases sexual stage-specific antigens such as Pfs48/45 and Pfs230 which have been previously shown to induce antibody responses (20, 44, 45).

Other described indicators of gametocyte carriage reproduced in our study are asexual parasitemia, age, and transmission intensity. Asexual parasitemia was a key predictor of gametocyte carriage in both cohorts as has been previously shown in Thailand (4), Papua New Guinea (22), and Kenya (18, 19). This strong association has been attributed to the fact that gametocytes are produced from a subset of asexual parasites (5) thus higher asexual parasitemia reflect increased odds of gametocyte carriage. This was probably why antibody responses to the asexual antigen AMA1 were associated with gametocytemia. We also found young host age to be predictive of gametocyte carriage as previously established (22, 24, 34). The association between younger children and gametocyte carriage has been attributed to the high asexual parasite densities observed in this age group (12). Transmission intensity was modestly predictive of gametocyte positivity as we observed a trend toward higher odds of gametocyte carriage during the rainy season when there is increased malaria transmission as has been previously reported (16, 34, 46). Moreover, residing in an area of low malaria transmission, as was the case for Ngerenya Late participants, showed less likelihood of gametocyte positivity further underlining the importance of transmission intensity (18). These well described indicators of gametocyte carriage, together with other screening tools, can thus provide a means to identify populations where malaria control efforts should be intensified and/or implemented.

## Conclusion

By exploring two independent study cohorts, we have demonstrated that ELISA-based IgG antibody responses to crude gametocyte extract are predictive of gametocytemia. However, anti-AMA1 antibodies were either a stronger independent predictor of gametocytemia or equally predictive as anti-gametocyte extract antibodies. We therefore conclude that IgG responses to crude gametocyte extract are not an independent predictor of gametocyte carriage, but rather a marker of more intense exposure to parasites which in turn makes gametocyte carriage more likely. In the absence of recombinant protein to asexual antigens, responses to gametocyte extract can serve as a serological screening tool to define the gametocyte and/or infectious reservoir. In addition, we did not observe significantly different levels of gametocytemia between the different age-groups, thus demonstrating that both children and adults contribute in malaria transmission. Therefore, this makes a case for broad application of transmission-blocking interventions across all age groups to have an impact on malaria transmission.

## Data Availability Statement

The datasets presented in this study can be found in online repositories. The name of the repository and accession number can be found here: Harvard Dataverse; https://doi.org/10.7910/DVN/UZWG9J.

## Ethics Statement

The studies involving human participants were reviewed and approved by the Kenya Medical Research Institute Ethics Review Committee. Written informed consent to participate in this study was provided by the participants’ legal guardian/next of kin.

## Author Contributions

BO, MM, and WM performed the experiments. BO, MM, and BO performed the statistical analysis. BO and MM wrote the paper. MK conceived and designed the study, and RM, TB, CD, KM, and PB provided advice. All authors contributed to the article and approved the submitted version.

## Funding

The AFIRM study was supported by a Bill and Melinda Gates Foundation grant (OPP1034789). The KMLC study was supported by a grant from the Wellcome Trust (092741). BO, MM, and WM are IDeAL scholars whose studentships are funded by DELTAS Africa Programme *via* the Wellcome Trust grant (107769) to KEMRI-Wellcome Trust Research Programme. PB and MK were supported by a Wellcome Trust grant (107499).

## Conflict of Interest

The authors declare that the research was conducted in the absence of any commercial or financial relationships that could be construed as a potential conflict of interest.
