# Supplementary material for: Antibody Responses to Crude Gametocyte Extract Predict Plasmodium falciparum Gametocyte Carriage in Kenya
Source: Front Immunol. 2021 Feb 3;11:609474. doi: 10.3389/fimmu.2020.609474 (PMC7902058; doi:10.3389/fimmu.2020.609474)
Supplement: Supplementary file 1 [file DataSheet_1.docx]

Supplementary Material

# Supplementary Figures and Tables

## Supplementary Figures

Supplementary Figure 1: Gametocyte culture at harvest. *P. falciparum* NF54 gametocytes were harvested on day 13 post induction when the parasites were between stage IV and V at 3% gametocytaemia. Arrow shows a stage V gametocyte. Scale bar: 20µm.

Supplementary Figure 2: Parasitaemia in the AFIRM cohort by age. Parasites were detected by either: microscopy (A) asexual parasites and (B) gametocytes or molecular assays (C) 18s QT NASBA (asexual parasites) and (D) Pfs25 QT NASBA (gametocytes). Box-whisker plots showing median, minimum and maximum with individual measurements represented by each dot. Wilcoxon test was used to test significance which is shown and was considered at *p*=0.05 where **p* < 0.05, ***p* < 0.01, ****p* <0.001, *****p* < 0.0001 and ns is not significant.

Supplementary Figure 3: Parasitaemia by season in the AFIRM cohort. Parasites were detected by either: microscopy (A) asexual parasites and (B) gametocytes or molecular assays (C) 18s QT NASBA (asexual parasites) and (D) Pfs25 QT NASBA (gametocytes). Box-whisker plots showing median, minimum and maximum with individual measurements represented by each dot. Wilcoxon test was used to test significance which is shown and was considered at *p*=0.05.

Supplementary Figure 4: Parasitaemia by age and transmission setting in KMLC cohort. Parasites were detected by microscopy. (A) Asexual parasites and (B) gametocytes by age. (C) Asexual parasites and (D) gametocytes by transmission setting. Box-whisker plots showing median, minimum and maximum with individual measurements represented by each dot. Wilcoxon test was used to test significance which is shown and was considered at *p*=0.05 where **p* < 0.05, ***p* < 0.01, ****p* <0.001, *****p* < 0.0001 and ns is not significant.

**Supplementary Figure 5: Correlation between parasitaemia and antibody response to crude gametocyte extract in the combined cohorts (AFIRM and KMLC)**. Parasitaemia was measured by microscopy while IgG antibody responses to crude gametocyte extract were measured by ELISA. Individual dots in the scatterplot and spearman correlation represent corresponding measurements from the same participant while regression line (blue) shows line of best fit and confidence intervals shown in grey.

## Supplementary Tables

Supplementary Table 1: Combined cohort (AFIRM & KMLC) – Predictors of microscopic gametocytaemia

|  | Univariable Analysis | | | Multivariable Analysis | | |
| --- | --- | --- | --- | --- | --- | --- |
| Covariate | **Odds ratio** | **95% C. I** | ***p value*** | **Odds ratio** | **95% C. I** | ***p value*** |
| Age group |  |  |  |  |  |  |
| < 5 years | 1.00 | - | - | 1.00 | - | - |
| 5 – 9 years | 2.12 | 1.21 - 3.71 | **0.008** | 2.49 | 1.28 - 4.85 | **0.007** |
| 10 – 15 years | 0.80 | 0.36 - 1.80 | 0.410 | 0.70 | 0.26 - 1.88 | 0.300 |
| > 15 years | 0.06 | 0.01 - 0.29 | **<0.001** | 0.09 | 0.02 - 0.43 | **0.002** |
| Asexual parasitaemia |  |  |  |  |  |  |
| Negative | 1.00 | - | **-** | 1.00 | - | **-** |
| Positive | 11.05 | 6.26 - 19.5 | **<0.001** | 6.70 | 3.30 - 13.58 | **<0.001** |
| Gametocyte extract response | 0.99 | 0.8 - 1.23 | 0.930 | 1.06 | 0.67 - 1.66 | 0.804 |
| AMA1 response | 1.38 | 1.07 - 1.79 | **0.012** | 1.30 | 0.90 - 1.88 | 0.150 |

Supplementary Table 2: AFIRM Cohort - Predictors of molecular gametocytaemia (adjusted for AMA1)

|  | Univariable Analysis | | | Multivariable Analysis | | |
| --- | --- | --- | --- | --- | --- | --- |
| Covariate | **Odds ratio** | **95% C. I** | ***p value*** | **Odds ratio** | **95% C. I** | ***p value*** |
| Age group |  |  |  |  |  |  |
| <5 years | 1.00 | - | - | 1.00 | - | - |
| 5 – 15 years | 2.72 | 1.36 - 5.44 | **0.005** | 2.37 | 0.99 - 5.66 | **0.051** |
| >15 years | 1.48 | 0.77 - 2.87 | 0.240 | 1.02 | 0.43 - 2.38 | 0.971 |
| Asexual parasitaemia |  |  |  |  |  |  |
| Negative | 1.00 | _ | **_** | 1.00 | - | **-** |
| Positive | 91.12 | 27.97 - 297.04 | **<0.001** | 70.77 | 21.44 - 233.53 | **<0.001** |
| AMA1 response | 5.18 | 3.24 - 8.27 | **<0.001** | 3.73 | 2.03 - 6.74 | **<0.001** |
| Season |  |  |  |  |  |  |
| Dry | 1.00 | - | **-** | 1.00 | - | - |
| Wet | 1.91 | 1.15- 3.17 | **0.012** | 1.11 | 0.52 - 2.37 | 0.794 |

Supplementary Table 3: KMLC Cohort – Predictors of microscopic gametocytaemia (adjusted for AMA1)

|  | Univariable Analysis | | | Multivariable Analysis | | |
| --- | --- | --- | --- | --- | --- | --- |
| Covariate | **Odds ratio** | **95% C. I** | ***p value*** | **Odds ratio** | **95% C. I** | ***p value*** |
| Age group |  |  |  |  |  |  |
| <5 years | 1.00 | - | - | 1.00 | - | - |
| 5-9 years | 1.25 | 0.66 - 2.37 | 0.490 | 0.62 | 0.20 - 1.96 | 0.412 |
| 10-15 years | 0.95 | 0.39 - 2.32 | 0.910 | 1.03 | 0.48 - 2.20 | 0.945 |
| Asexual parasitaemia | | | | | | |
| Negative | 1.00 | - | - | 1.00 | - | - |
| Positive | 5.80 | 2.98 - 11.28 | **<0.001** | 1.33 | 0.55 - 3.21 | 0.517 |
| AMA1 response | 2.06 | 1.56 - 2.73 | **<0.001** | 1.61 | 1.08 - 2.42 | **0.020** |
| Cohort |  |  |  |  |  |  |
| Junju | 1.00 | - | - | 1.00 | - | - |
| Ngerenya Early | 2.03 | 0.97 - 4.28 | **0.061** | 1.68 | 0.76 - 3.70 | 0.197 |
| Ngerenya Late | 0.13 | 0.05 - 0.32 | **<0.001** | 0.18 | 0.06 - 0.44 | **<0.001** |
