# Supplementary figures and images for: Antibody Responses to Crude Gametocyte Extract Predict Plasmodium falciparum Gametocyte Carriage in Kenya
Source: Front Immunol. 2021 Feb 3;11:609474. doi: 10.3389/fimmu.2020.609474 (PMC7902058; doi:10.3389/fimmu.2020.609474)

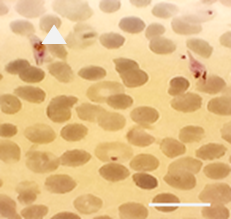

Supplement: Supplementary file 2 [file Image_1.tif]

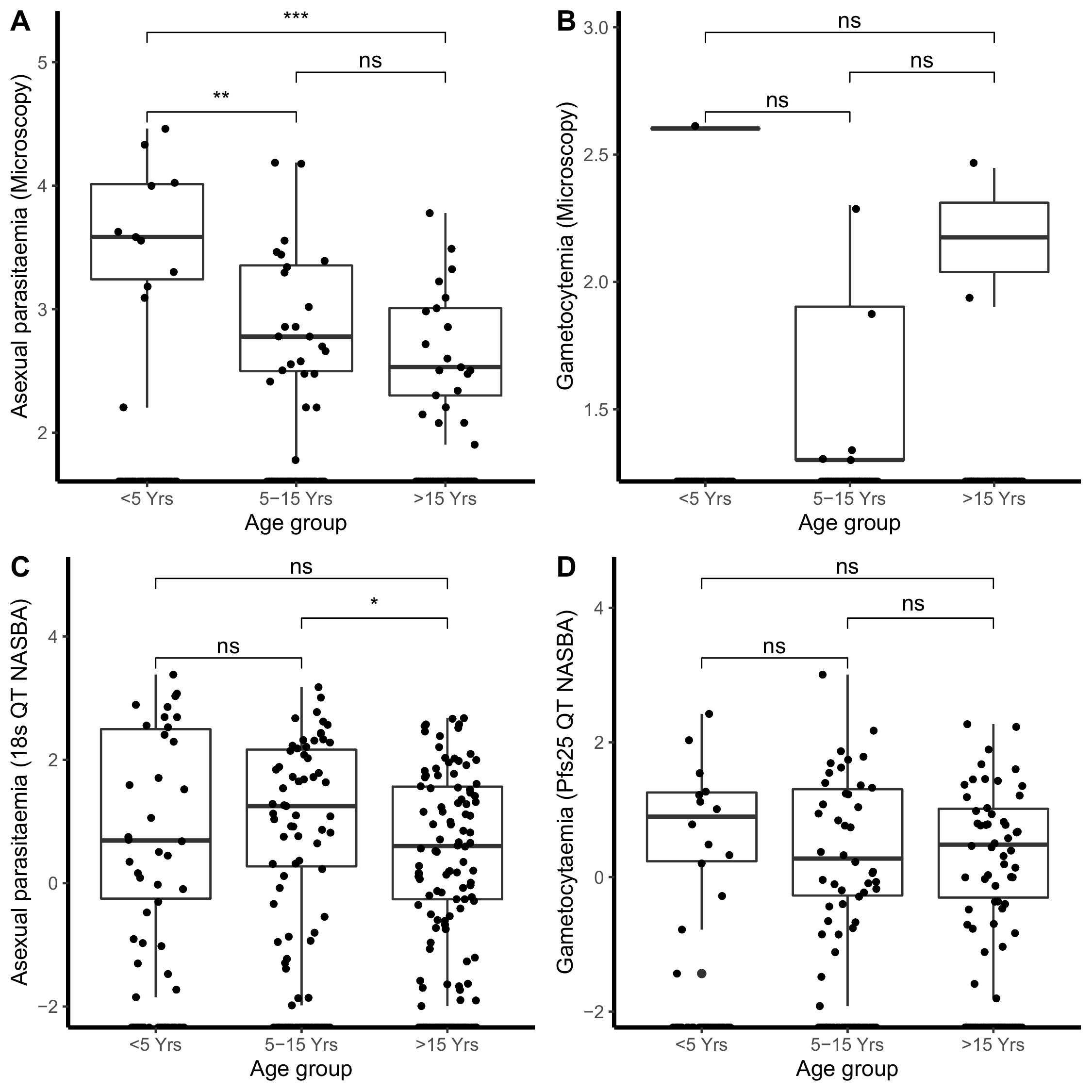

Supplement: Supplementary file 3 [file Image_2.tif]

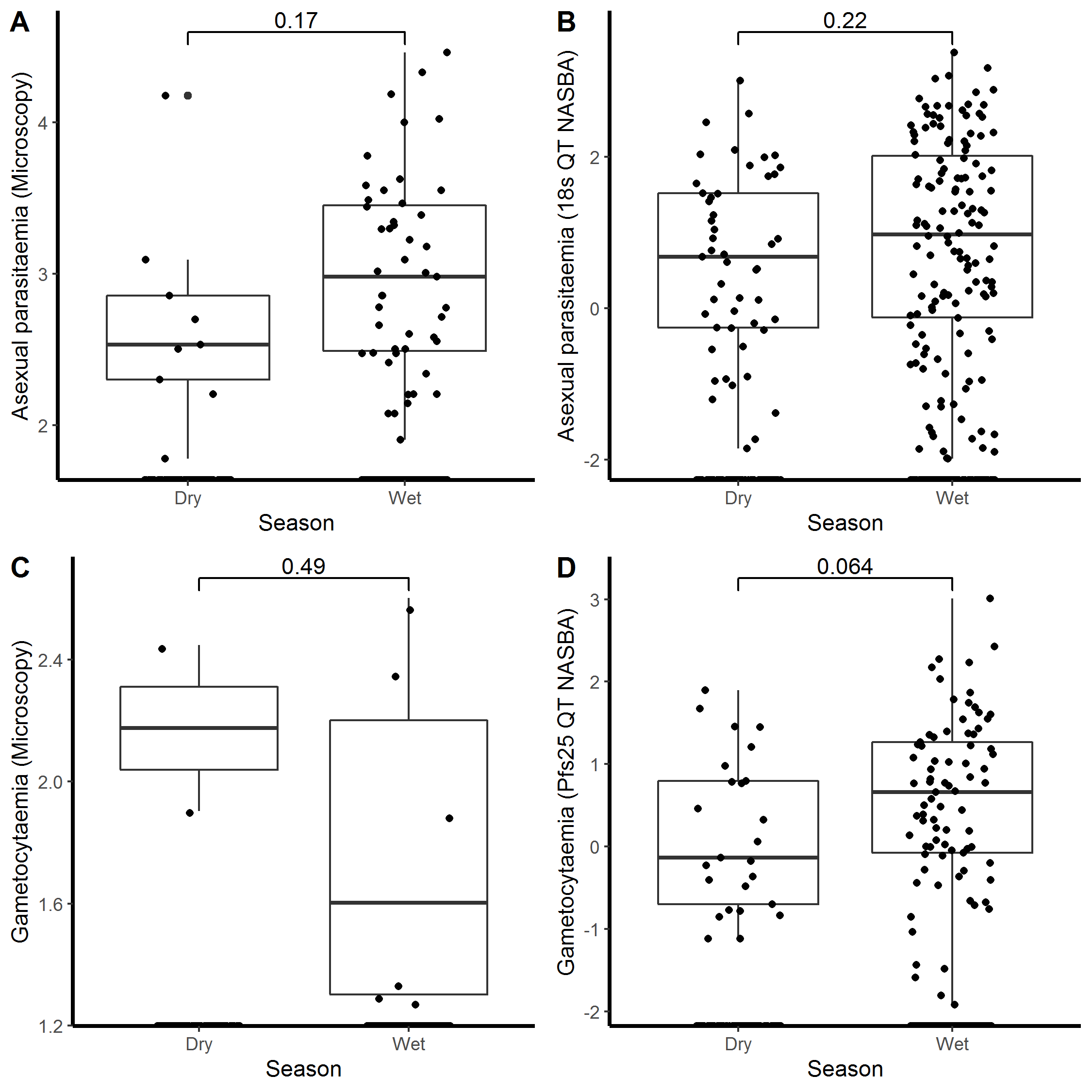

Supplement: Supplementary file 4 [file Image_3.tiff]

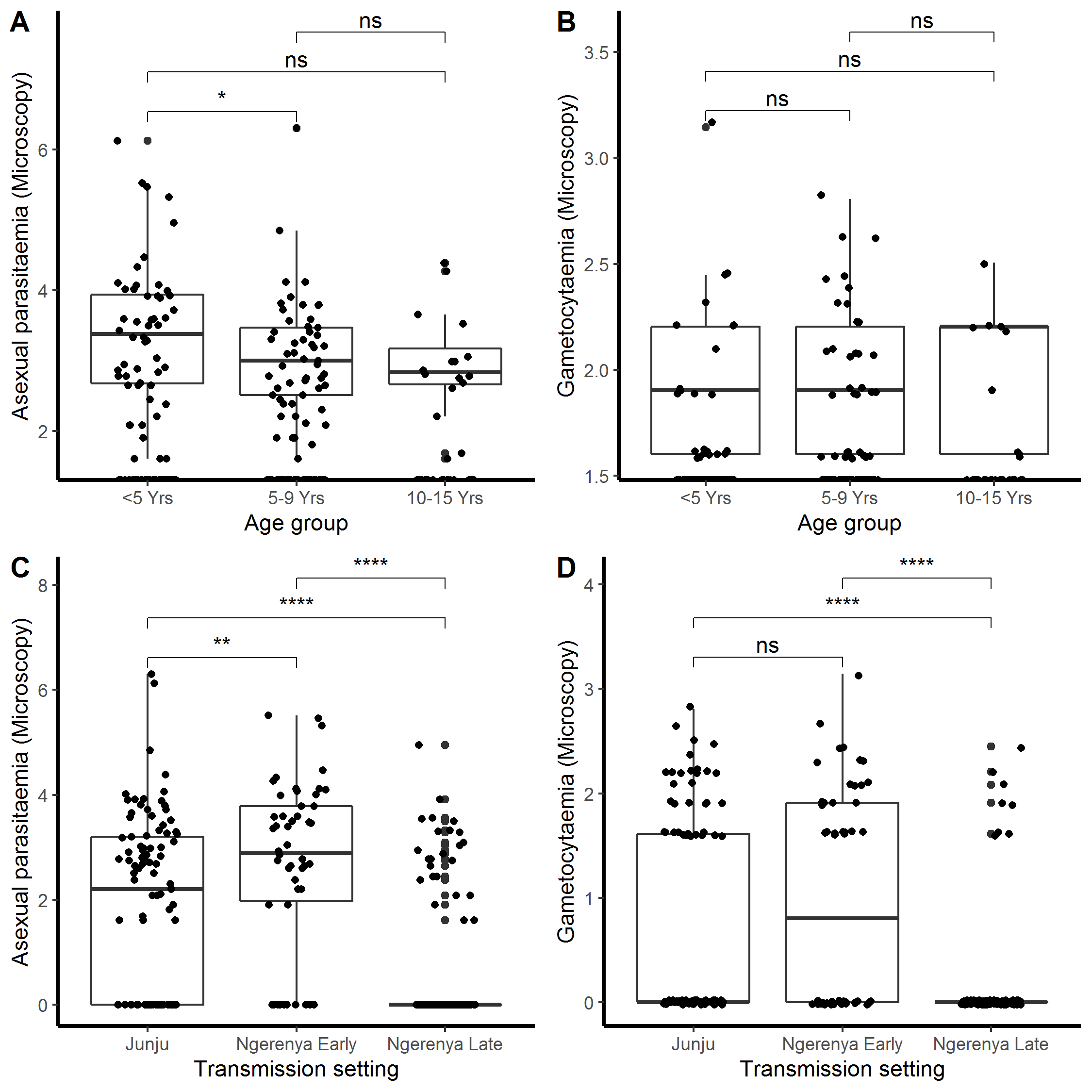

Supplement: Supplementary file 5 [file Image_4.tiff]

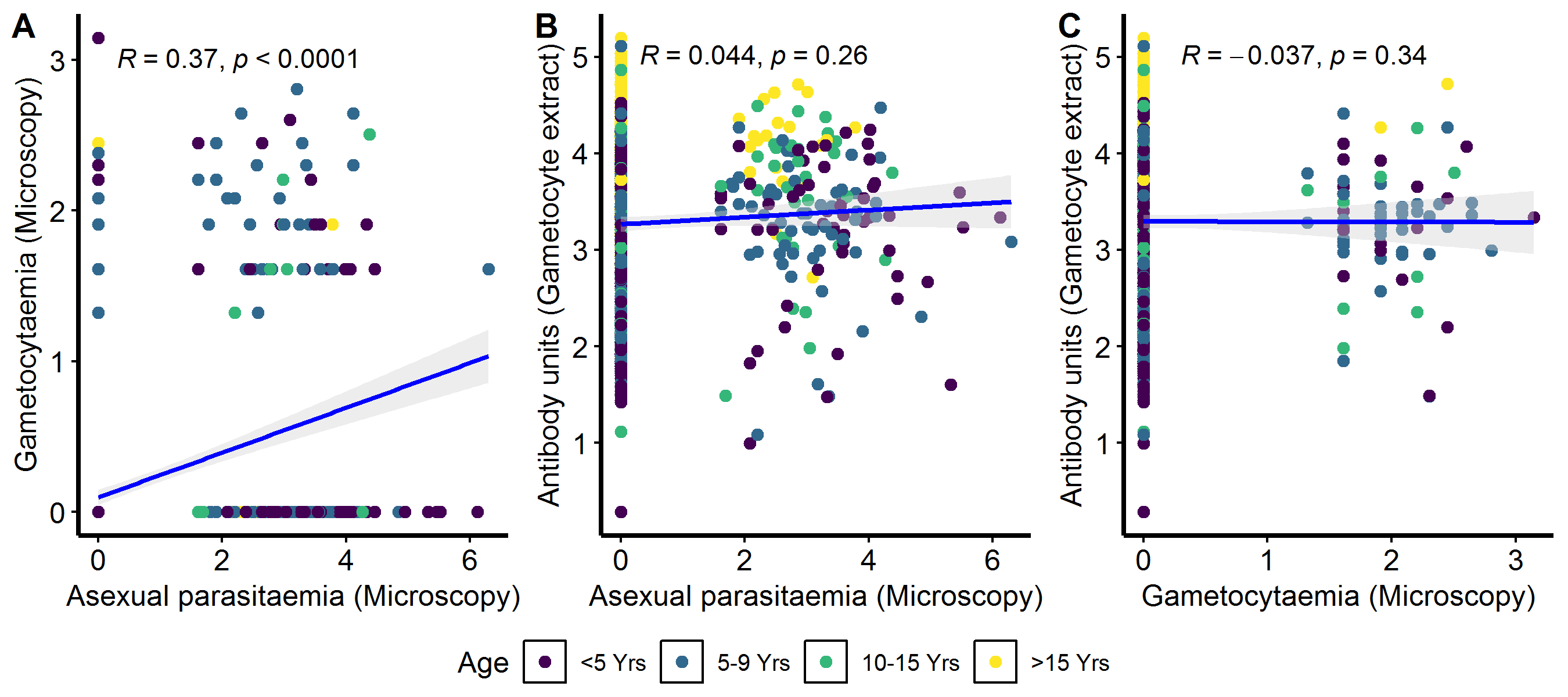

Supplement: Supplementary file 6 [file Image_5.tiff]
